# Assessing the Correlation Between Inter-Leg Ankle–Brachial Index Asymmetry and Peripheral Artery Disease Outcomes

**DOI:** 10.3390/jcdd13090470

**Published:** 2026-09-17

**Authors:** Ben Li, Mariam Zakaria, Mo’ath Bani Ali, Ola Kassem, Houssam Younes, Abdelrahman Zamzam, Nadya Al Matrooshi, Rawand Abdin, Mohammad Qadura

**Affiliations:** 1 Division of Vascular Surgery, St. Michael’s Hospital, Unity Health Toronto, University of Toronto, Toronto, ON M5B 1W8, Canada; benx.li@mail.utoronto.ca (B.L.);; 2 Department of Surgery, University of Toronto, Toronto, ON M5S 1A1, Canada; 3 Temerty Centre for Artificial Intelligence Research and Education in Medicine (T-CAIREM), University of Toronto, Toronto, ON M5S 1A1, Canada; 4 Institute of Medical Science, University of Toronto, Toronto, ON M5S 1A1, Canada; 5 Heart, Vascular, & Thoracic Institute, Cleveland Clinic Abu Dhabi, Abu Dhabi 112412, United Arab Emirates; 6 Department of Medicine, McMaster University, Hamilton, ON L8S 4L8, Canada; 7 Li Ka Shing Knowledge Institute, St. Michael’s Hospital, Unity Health Toronto, University of Toronto, Toronto, ON M5B 1W8, Canada

**Keywords:** ankle–brachial index, asymmetry, inter-leg, prognosis, peripheral artery disease

## Abstract

Background/Objectives: Peripheral artery disease (PAD) remains underdiagnosed and suboptimally treated, with limited disease awareness and underutilization of available diagnostic strategies representing important contributing factors. The ankle–brachial index (ABI) is the gold-standard screening test for PAD and has been shown to correlate with limb outcomes. However, the current interpretation of ABIs does not account for clinically significant differences between legs, which may reflect asymmetric disease burden and have an impact on limb outcomes. The association between inter-leg ABI asymmetry and PAD outcomes is unclear and requires further study. Thus, we assessed the prognostic importance of a clinically significant difference in left and right ABI values (≥0.15) on limb outcomes in patients with PAD. Methods: A total of 3313 patients with PAD who received outpatient vascular care at a single institution were retrospectively analyzed. At baseline, ABIs were measured in a certified vascular laboratory by dividing the higher of the dorsalis pedis or posterior tibial artery systolic pressures in each leg by the higher brachial systolic pressure. Inter-leg ABI asymmetry was defined by an absolute difference between left and right ABI values ≥ 0.15, which has been shown to be clinically significant. The primary outcome was major adverse limb event (MALE) over 2 years of follow-up. MALE was defined as the need for vascular intervention (either open or endovascular lower extremity revascularization) or major lower extremity amputation above the ankle. The association between inter-leg ABI asymmetry and 2-year MALE was evaluated using univariable and multivariable logistic regression models adjusted for age, sex, body mass index (BMI), and ABI-defined hemodynamic severity. Results: The mean participant age was 58.4 years (SD 12.9), 966 (29.2%) participants were female, and the mean BMI was 28.8 kg/m^2^ (SD 5.7). There were 666 (20.1%) patients who had inter-leg ABI asymmetry. The mean inter-leg ABI difference was 0.38 (SD 0.29) in the inter-leg ABI asymmetry group and 0.04 (SD 0.02) in the no inter-leg ABI asymmetry group (*p* < 0.001). Patients with inter-leg ABI asymmetry were more likely to have moderate PAD (36.0% vs. 6.5%, *p* < 0.001) and severe PAD (6.2% vs. 0.7%, *p* < 0.001), and less likely to have mild PAD (57.8% vs. 92.8%, *p* < 0.001), compared to patients with no inter-leg ABI asymmetry. Over the median follow-up period of 2 years, 304 (9.2%) participants experienced MALE. The 2-year MALE rate was significantly higher in patients with inter-leg ABI asymmetry compared to those without (26.0% vs. 4.9%, *p* < 0.001). Similarly, patients with inter-leg ABI asymmetry had a higher rate of the need for revascularization (21.5% vs. 4.1%, *p* < 0.001) and major amputation (10.2% vs. 1.8%, *p* < 0.001). Multivariable logistic regression controlling for age, sex, BMI, and ABI-defined hemodynamic severity showed that inter-leg ABI asymmetry was independently associated with 2-year MALE (adjusted OR 3.70, 95% CI 2.80–4.91, *p* < 0.001). Conclusions: In this study, over 20% of patients with PAD had clinically significant inter-leg ABI asymmetry, which was independently associated with a nearly four times higher odds of 2-year MALE, including the need for revascularization and major amputation. Our results suggest that inter-leg ABI asymmetry may represent a readily available prognostic marker of adverse limb outcomes in patients with PAD. Further external validation and prospective studies are needed to determine whether incorporating inter-leg ABI asymmetry into clinical assessment improves risk stratification or clinical outcomes.

## 1. Introduction

Peripheral artery disease (PAD) affects more than 200 million individuals globally [1,2]. Despite being a significant contributor to limb loss and death, PAD is often underdiagnosed and suboptimally treated [3]. Important contributing factors include limited awareness of PAD among healthcare providers and patients, as well as underutilization of available diagnostic strategies [3].

The ankle–brachial index (ABI) remains the gold-standard screening tool for PAD [4]. It is calculated by measuring systolic blood pressure at the ankle in both the dorsalis pedis (DP) and posterior tibial (PT) arteries [4]. For each leg, the higher of these two ankle pressures is then divided by the higher brachial systolic pressure [4]. This produces a single ABI value per limb, which is used both for PAD screening (ABI < 0.9) and for grading disease severity as mild (0.7–0.89), moderate (0.4–0.69), or severe (<0.4) [5]. Another important limitation of ABI is that medial arterial calcification and noncompressible ankle arteries, particularly in patients with diabetes, can result in falsely elevated ABI measurements and reduce its diagnostic reliability [4].

While lower ABIs have been demonstrated to be associated with adverse PAD outcomes [6], there has been limited study on the correlation between clinically significant inter-leg ABI asymmetry ≥ 0.15 and adverse limb events [5,7]. Inter-leg ABI asymmetry may be associated with worse PAD outcomes because it suggests the potential for uneven plaque distribution in the lower extremity vasculature leading to differences in arterial perfusion between limbs [8,9,10,11]. This may reflect more severe disease in localized vessels in either the left or right leg, increasing the potential for the need for surgical revascularization or amputation of the affected limb [8,9,10,11]. Inter-leg ABI asymmetry may reflect underlying differences in arterial perfusion between the lower extremities resulting from an unequal distribution of arterial stenoses or occlusions. Other vascular processes, including thrombosis of individual vessels, may also contribute to asymmetric perfusion. Thus, inter-leg ABI asymmetry may serve as a marker of underlying differences in lower extremity arterial disease rather than directly contributing to adverse limb outcomes.

To further understand this problem and to address this clinical gap, we evaluated the clinical significance of discrepancies in inter-leg ABI measurements. Specifically, we assessed whether a clinically relevant discordance of ≥0.15 between the left and right ABI values has prognostic value for major adverse limb events (MALEs) in patients with PAD [5,7]. Our hypothesis was that patients with inter-leg ABI asymmetry have uneven plaque burden that puts them at an increased risk of adverse limb outcomes. If confirmed, our hypothesis would underscore the clinical importance of incorporating inter-leg ABI asymmetry into the evaluation of patients with PAD, which may enhance risk stratification and inform subsequent diagnostic workup and management.

## 2. Materials and Methods

### 2.1. Ethical Approval

Ethics approval for this study was granted by the Institutional Review Board of Cleveland Clinic Abu Dhabi on 1 March 2023 (IRB # A-2023-033). Informed consent for participation was not required, as per local legislation by the Institutional Review Board of Cleveland Clinic Abu Dhabi, given that this was a retrospective analysis of anonymized data. The study procedures adhered to the ethical standards outlined in the Declaration of Helsinki [12].

### 2.2. Study Design

This investigation was conducted as a retrospective cohort study, and results reported in accordance with the Strengthening the Reporting of Observational Studies in Epidemiology (STROBE) guidelines [13].

### 2.3. Patient Recruitment

Between March 2018 and February 2022, patients with PAD who attended outpatient vascular clinics at our hospital were retrospectively analyzed. PAD was defined as an ABI ≤ 0.9 or a toe brachial index (TBI) ≤ 0.67, together with absent or diminished pedal pulses, in at least 1 leg [14]. ABI measurements were obtained from both lower extremities as part of the same baseline vascular laboratory assessment. Detailed information regarding resting time, measurement order, and repeated measurements was not available in the retrospective dataset, although all measurements were performed in a certified vascular laboratory using standardized vascular laboratory procedures. TBI was used when ABI measurements were considered unreliable because of noncompressible vessels [14]. Exclusion criteria included acute limb ischemia, acute coronary syndrome, or elevated troponin within the preceding three months. Patients without ABI measurements in both legs were excluded.

### 2.4. Baseline Patient Characteristics

Baseline demographic characteristics were obtained for all participants, including age, sex, and body mass index (BMI). ABIs were measured in a certified vascular laboratory by dividing the higher of the dorsalis pedis (DP) or posterior tibial (PT) artery systolic pressures in each leg by the higher brachial systolic pressure [4]. Inter-leg ABI asymmetry was defined a priori as an absolute difference of ≥0.15 between the left and right leg ABI values based on the prior literature [5,7].

### 2.5. Follow-Up and Outcomes

Outpatient clinic visits were scheduled at 1 year and 2 years post-baseline assessment. The primary outcome was a composite MALE outcome over the 2-year follow-up period, defined as major lower extremity amputation above the ankle or the need for open or endovascular lower extremity revascularization. The individual components of the composite outcome were also evaluated separately.

### 2.6. Statistical Analysis

Statistical analyses were conducted at the patient level. Continuous variables were summarized as mean (standard deviation; SD) and median [interquartile range; IQR], and categorical variables as counts (%). Baseline characteristics were compared between participants with and without inter-leg ABI asymmetry using the independent-samples t test for normally distributed variables and the Wilcoxon rank-sum test for non-normally distributed variables. Categorical variables were compared using the chi-square test or Fisher’s exact test when expected cell counts were small (<5). Inter-leg ABI asymmetry was defined using ABI measurements derived from the left and right legs of the same patient, with asymmetry classified as present when the absolute difference between the left and right leg ABI values was ≥0.15. Associations between inter-leg ABI asymmetry and clinical outcomes were evaluated using logistic regression models to estimate odds ratios (ORs) with 95% confidence intervals (CIs). Multivariable models adjusted for age, sex, BMI, and ABI-defined hemodynamic severity (mild [0.7–0.89], moderate [0.4–0.69], or severe [<0.4]) [5]. ABI-defined hemodynamic severity was determined using the lower of the left and right ABI values for each patient, representing the more hemodynamically compromised limb [4]. Logistic regression was used because the primary analysis evaluated the occurrence of adverse limb outcomes within a prespecified 2-year follow-up period rather than the time to occurrence of these events. All tests were two-sided, and statistical significance was set at *p* < 0.05. Python version 3.13.3 was used for all statistical analyses [15].

## 3. Results

### 3.1. Patients

The overall cohort included 3313 patients; 666 (20.1%) had inter-leg ABI asymmetry and 2647 (79.9%) did not. Mean age was 56.9 (SD 13.1) years in the no inter-leg asymmetry group, and 64.4 (SD 12.2) years in the inter-leg asymmetry group, *p* < 0.001. There were 789 (29.8%) female patients in the no inter-leg asymmetry group and 177 (26.6%) in the inter-leg asymmetry group, *p* = 0.10. Mean BMI was 28.8 (SD 5.8) kg/m^2^ in the no inter-leg asymmetry group and 28.7 (SD 5.5) kg/m^2^ in the inter-leg asymmetry group, *p* = 0.69. The mean inter-leg ABI difference was 0.04 (SD 0.02) in the no inter-leg asymmetry group and 0.38 (SD 0.29) in the inter-leg asymmetry group, *p* < 0.001 (Table 1). All patients included in the final analytic cohort had the required bilateral ABI measurements, covariate data, and 2-year outcome data; therefore, no additional patients were excluded from the analytic cohort because of missing follow-up or covariate data.

### 3.2. Severity of PAD in Patients with and Without Inter-Leg ABI Asymmetry

Patients with inter-leg ABI asymmetry were more likely to have moderate PAD (36.0% vs. 6.5%, *p* < 0.001) and severe PAD (6.2% vs. 0.7%, *p* < 0.001) and less likely to have mild PAD (57.8% vs. 92.8%, *p* < 0.001) compared to patients with no inter-leg ABI asymmetry. This finding suggests that patients with inter-leg ABI asymmetry are more likely to have more advanced PAD (Table 2).

### 3.3. Events

Overall, 2-year MALE occurred in 304 (9.2%) patients, and the event rate was significantly higher in patients with inter-leg asymmetry compared to those without (26.0% vs. 4.9%, *p* < 0.001). Two-year major amputation occurred in 116 (3.5%) patients and was significantly higher in patients with inter-leg asymmetry compared to those with no inter-leg asymmetry (10.2% vs. 1.8%, *p* < 0.001). Similarly, 2-year need for revascularization occurred in 251 (7.6%) patients and was significantly higher in the inter-leg asymmetry group compared to the no inter-leg asymmetry group (21.5% vs. 4.1%, *p* < 0.001). These results suggest that adverse limb events occur more frequently in patients with inter-leg ABI asymmetry (Table 3).

### 3.4. Multivariable Analysis of the Association Between Inter-Leg ABI Asymmetry and Adverse Limb Events

In multivariable logistic regression models adjusted for age, sex, BMI, and ABI-defined hemodynamic severity, inter-leg asymmetry was a strong independent predictor across all outcomes. For 2-year major amputation, inter-leg asymmetry had an adjusted OR of 4.44 (95% CI, 2.88–6.84; *p* < 0.001). For 2-year need for revascularization, inter-leg asymmetry had an adjusted OR of 3.06 (95% CI, 2.25–4.16; *p* < 0.001). Similarly, in the model predicting 2-year MALE, inter-leg asymmetry had an adjusted OR of 3.70 (95% CI, 2.80–4.91; *p* < 0.001). These results suggest that inter-leg ABI asymmetry was associated with a nearly 4-fold higher odds of developing 2-year MALE (Table 4).

## 4. Discussion

### 4.1. Summary of Findings

In this study, we showed several key findings. First, over 20% of patients with PAD had inter-leg ABI asymmetry, defined as an absolute difference between left and right ABI values ≥ 0.15. This suggests the potential for uneven distribution of plaque burden in a sizable proportion of the PAD population [8,9,10,11]. Second, patients with inter-leg ABI asymmetry were more likely to have moderate or severe PAD compared to patients without inter-leg ABI asymmetry. Thus, inter-leg asymmetry may be a marker of more advanced disease [8,9,10,11]. Third, patients with inter-leg ABI asymmetry were more likely to develop MALE over 2 years of follow-up compared to patients without inter-leg ABI asymmetry (26.0% vs. 4.9%), including both the need for revascularization (21.5% vs. 4.1%) and major amputation (10.2% vs. 1.8%). Multivariable analysis confirmed an independent association between inter-leg ABI asymmetry and 2-year MALE (adjusted OR 3.70). This represents a nearly 4-fold increased odds of 2-year MALE in patients with inter-leg ABI asymmetry compared to those without. Importantly, the association with inter-leg ABI asymmetry was observed for both components of the composite outcome. The particularly strong association with major amputation (adjusted OR 4.44) supports the prognostic signal observed in the composite analysis, as major amputation represents a clinically important limb outcome that may be less influenced by variation in clinical decision-making and practice patterns than revascularization. Our findings suggest that inter-leg ABI asymmetry is an important prognostic marker for adverse limb events and should be taken into consideration by clinicians for PAD screening, risk stratification, and treatment decision making. Additionally, further basic, translational, and clinical research is needed to understand the pathophysiology underpinning the association between inter-leg ABI asymmetry and adverse limb events, with the goal of supporting more accurate risk-stratification, personalized medical management, and improved outcomes in patients with PAD.

### 4.2. Comparison to the Existing Literature

While the prognostic importance of ABI for PAD outcomes has been previously demonstrated [6], no previous work has investigated the correlation between inter-leg ABI asymmetry and adverse limb events. Secchi et al. (2020) employed magnetic resonance angiography to evaluate symmetry of lower extremity arterial disease in patients with PAD, demonstrating that asymmetry between limbs can occur; however, they did not examine inter-leg differences in ABI or their relationships to clinical outcomes [8]. Pacha et al. (2018) reported that individuals with an ABI < 0.5 had a 2.7-fold increased likelihood of undergoing surgical revascularization compared to those with ABI > 0.5, though inter-leg ABI discordance was not evaluated [16]. Lee and colleagues (2023) found that Black adults with abnormal ABI values had approximately double the risk of major adverse cardiovascular events and mortality relative to those with normal ABI, but did not assess inter-leg variation [17]. McDermott and colleagues demonstrated a strong association between ABI and functional status, with only 40% of patients with ABI < 0.4 able to walk continuously for six minutes, compared with over 95% among those with ABI values between 1.00 and 1.50 [18]. Similarly, Paskiewicz et al. (2021) showed that an ABI ≤ 0.90, compared with 1.11–1.20, was associated with an approximately four-fold increased risk of chronic limb-threatening ischemia and ischemic amputation [19]. Lin et al. (2015) identified an inter-leg ABI difference ≥ 0.15 as an independent predictor of mortality in patients receiving hemodialysis, although limb-specific PAD outcomes were not assessed [20]. Our study adds to this evidence by showing that not only are single ABI measurements important in predicting PAD outcomes, but that inter-leg ABI asymmetry also has significant prognostic value for adverse limb events to guide clinical decision making. Collectively, these observations highlight the need for additional mechanistic research to better define the biological processes linking inter-leg ABI asymmetry with PAD progression. Elucidating these pathways may enhance risk stratification and inform the development of more targeted strategies aimed at preserving limb function and optimizing clinical outcomes in patients with PAD.

### 4.3. Explanation of Findings

There are several explanations for our findings. First, over 20% of patients in our cohort had inter-leg ABI asymmetry. This is likely because atherosclerotic disease can affect the right and left legs differently. In a study of 12,312 consecutive patients at Mayo Clinic, Cohoon and colleagues (2017) showed that the prevalence of PAD based on an ABI ≤ 0.90 was significantly higher on the left leg than the right leg [9]. Similarly, Hiatt and colleagues studied over 1000 patients and showed that the mean left leg ABI was 0.02 lower than the right leg ABI [11]. Smith et al. (2003) also showed that, in 695 patients, the mean ABI was 0.03 higher in the right leg than the left leg [10]. The reasons for these findings are unclear but may be related to the order with which the ABIs are measured in the vascular laboratory (usually right before left), the location of the aorta relative to the lower extremity vasculature (the aorta generally lies towards the left side of the abdomen), or other anatomic, physiologic, or technical factors [5]. Further basic and translational work is needed to understand the reasons that contribute to inter-leg ABI asymmetry [5]. Second, patients with inter-leg ABI asymmetry were more likely to have more severe PAD. Inter-leg ABI asymmetry may reflect differences in arterial perfusion between the lower extremities resulting from an unequal distribution of atherosclerotic stenoses or occlusions. However, other vascular processes, including thrombosis or thromboembolism, may also contribute to asymmetric perfusion and inter-leg differences in ABI. Furthermore, plaque burden and distribution were not directly assessed in this study, and other anatomical, physiological, and technical factors may also contribute to inter-leg differences in ABI. Third, patients with inter-leg ABI asymmetry were more likely to have adverse limb events. This may be because inter-leg asymmetry may be a marker of more advanced PAD, increasing the risk for the need for revascularization and limb loss [5]. Notably, inter-leg ABI asymmetry remained significantly associated with 2-year MALE after controlling for ABI-defined hemodynamic severity, suggesting a potential mechanistic link beyond disease severity. Inter-leg ABI asymmetry should be interpreted as a marker of differences in hemodynamic compromise between the lower extremities rather than as a distinct pathological process. Its association with subsequent limb outcomes may reflect greater underlying arterial disease burden in one limb. Accordingly, the present findings support the potential prognostic relevance of inter-leg ABI asymmetry but do not establish a role for this measure as an independent diagnostic criterion for PAD. Patients with inter-leg ABI asymmetry were older than those without asymmetry, which may reflect greater cumulative and asymmetric atherosclerotic disease burden with increasing age. However, inter-leg ABI asymmetry remained independently associated with adverse limb outcomes after adjustment for age. Our findings require further confirmation through dedicated basic science and translational studies. Importantly, further work is needed to understand the pathophysiology underpinning the association between inter-leg ABI asymmetry and PAD outcomes to guide more accurate risk stratification, personalized medical management, and improved outcomes in patients with PAD [5].

### 4.4. Implications

Our findings have meaningful implications for the evaluation and management of patients with PAD. Our results provide evidence supporting the prognostic importance of inter-leg ABI asymmetry in relation to limb outcomes. Thus, we recommend assessing for inter-leg ABI asymmetry ≥ 0.15 in the workup of patients with PAD. If an inter-leg ABI asymmetry is detected, the patient may benefit from further workup including arterial duplex ultrasound to assess blood flow and to evaluate disease presence, location, and severity [21]. This may increase detection of uneven distribution of plaque burden, and these patients may benefit from additional vascular imaging including computed tomography angiography to evaluate anatomy and disease extent [22], which may support surgical planning in patients found to have advanced disease [23,24]. Inter-leg ABI asymmetry may reflect differences in arterial perfusion between the lower extremities resulting from an unequal distribution of atherosclerotic stenoses or occlusions. However, other vascular processes, including thrombosis or thromboembolism, may also contribute to asymmetric perfusion and inter-leg differences in ABI. Given that patients with inter-leg ABI asymmetry are at increased risk for adverse limb events, they may also benefit from aggressive risk reduction medications including antiplatelets, statins, and lifestyle modifications, as well as close follow-up to regularly assess their limb perfusion and reduce their risk of limb loss [25]. This approach may improve PAD management by allowing more accurate risk stratification and facilitating earlier therapeutic intervention [26]. In turn, it has the potential to enhance clinical outcomes while reducing overall healthcare utilization and costs [26]. Our findings suggest that inter-leg ABI asymmetry may provide additional prognostic information when interpreting ABI measurements in patients with PAD. Given that inter-leg ABI asymmetry can be derived from routinely obtained bilateral ABI measurements without additional testing, its potential role as a simple adjunctive prognostic marker warrants further investigation. Importantly, the present observational study was not designed to determine whether inter-leg ABI asymmetry should alter diagnostic testing, surveillance, or treatment decisions. Importantly, external validation of these findings is needed to assess the effectiveness and practicality of adopting this strategy in routine clinical practice.

The present findings demonstrate a patient-level association between inter-leg ABI asymmetry and subsequent adverse limb outcomes; however, future studies incorporating clinical symptom staging and limb-specific outcomes are needed to further characterize this relationship and determine the prognostic significance of inter-leg ABI asymmetry at the individual-limb level. The observed association with major amputation further supports prospective evaluation and external validation of this readily available measure. Although inter-leg ABI asymmetry remained independently associated with adverse limb outcomes after adjustment for ABI-defined hemodynamic severity based on the lower ABI, the present study did not evaluate the lower ABI and inter-leg ABI difference as continuous predictors or formally assess incremental predictive performance. Therefore, whether inter-leg ABI asymmetry provides additional prognostic information beyond the continuous lower ABI requires further investigation in appropriately designed prospective studies incorporating comprehensive clinical risk factors.

### 4.5. Limitations

Although these findings provide important insights into PAD assessment and risk stratification, several limitations should be considered. The study was performed at a single academic centre, which may limit the generalizability of the results to other populations. Accordingly, multicentre studies are needed to validate and extend these observations. Although the sample size of 3313 patients is substantial, larger cohorts in future investigations would enable more robust and externally generalizable analyses. Further imaging and translational work is needed to better understand the mechanisms driving inter-leg ABI asymmetry and its relationship with adverse limb outcomes. In addition, feasibility and cost-effectiveness studies will be important to evaluate the practical implementation of routine reporting and interpretation of inter-leg ABI differences in clinical practice. Detailed cardiovascular risk factors, comorbidities, prior vascular interventions, and medical therapies were not available in this retrospective dataset. Consequently, the multivariable analyses could not adjust for potentially important confounders including diabetes, smoking, chronic kidney disease, cardiovascular and cerebrovascular disease, hypertension, dyslipidemia, previous lower extremity interventions, and medical therapy, and residual confounding cannot be excluded.

Furthermore, clinical PAD severity according to Rutherford or Fontaine classification was not available in this retrospective dataset; therefore, ABI categories were used to characterize hemodynamic disease burden rather than clinical disease stage. Additionally, the laterality of subsequent revascularization and major amputation was not linked to baseline limb-specific ABI measurements. Therefore, we could not determine the relationship between clinical symptom severity, the limb with greater baseline hemodynamic impairment, and subsequent limb-specific outcomes. The composite MALE definition included the need for lower extremity revascularization, which represents a therapeutic intervention rather than an adverse outcome itself, although the requirement for intervention may reflect clinically significant progression of limb disease. Minor amputations were not captured as part of the prespecified outcome definition. Importantly, major amputation and revascularization were also analyzed separately. Although the composite limb outcome included both open and endovascular lower extremity revascularization, the specific type and laterality of individual revascularization procedures were not available for this analysis, limiting more detailed assessment of the relationship between baseline limb-specific ABI measurements and subsequent interventions.

The analysis was conducted at the patient level, and the laterality of subsequent revascularization and major amputation was not linked to baseline limb-specific ABI measurements. Therefore, we could not determine whether adverse limb events occurred preferentially in the limb with the lower baseline ABI. Future limb-level analyses are needed to characterize the relationship between the side of greater hemodynamic impairment and subsequent limb-specific outcomes. Although inter-leg ABI asymmetry remained independently associated with adverse limb outcomes after adjustment for ABI-defined hemodynamic severity, the present analysis did not specifically evaluate whether inter-leg ABI asymmetry provides prognostic information beyond the lowest ABI modeled as a continuous measure. Future studies should directly compare these measures and evaluate their incremental prognostic value.

Inter-leg ABI asymmetry was evaluated using a prespecified dichotomous threshold of ≥0.15. The present study did not evaluate inter-leg ABI difference as a continuous predictor or assess alternative thresholds; therefore, future studies should examine the dose–response relationship between increasing inter-leg ABI difference and adverse limb outcomes and determine whether alternative thresholds provide additional prognostic information. The present study evaluated the occurrence of outcomes over a prespecified 2-year follow-up period using logistic regression and did not evaluate time to event or competing risks. Future studies incorporating detailed time-to-event and mortality data may further characterize the relationship between inter-leg ABI asymmetry and adverse limb outcomes.

The present study evaluated the independent association between inter-leg ABI asymmetry and adverse limb outcomes but did not assess incremental predictive performance, calibration, or clinical utility. Future studies incorporating comprehensive clinical risk factors and conventional ABI measurements should determine whether inter-leg ABI asymmetry improves prediction beyond established risk factors and ABI measures. History of previous lower extremity revascularization and other vascular interventions was not available and could not be accounted for in the analysis, representing a potential source of residual confounding. Although ABI-defined hemodynamic severity was determined using the lower ABI, the distributions of the individual left and right, lowest, and highest ABI measurements were not separately reported, limiting more detailed characterization of the relationship between absolute ABI values and inter-leg asymmetry.

## 5. Conclusions

In this study, over 20% of patients had inter-leg ABI asymmetry (absolute difference in left and right ABI values ≥ 0.15), which was independently associated with nearly 4-fold higher odds of 2-year MALE, including the need for revascularization and major amputation. These findings suggest that inter-leg ABI asymmetry may represent a readily available prognostic marker for adverse limb events in patients with PAD. However, external validation and prospective studies are needed to determine whether incorporating inter-leg ABI asymmetry into clinical risk assessment provides incremental prognostic value and improves clinical decision making or patient outcomes.

## Figures and Tables

**Table 1:** Baseline characteristics.

	No Inter-Leg ABI Asymmetry (*n* = 2647)	Inter-Leg ABI Asymmetry (*n* = 666)	*p* Value
Age (years), mean (SD)	56.9 (13.1)	64.4 (12.2)	<0.001
Female, *n* (%)	789 (29.8%)	177 (26.6%)	0.10
BMI (kg/m^2^), mean (SD)	28.8 (5.8)	28.7 (5.5)	0.69
Inter-leg ABI difference, mean (SD)	0.04 (0.02)	0.38 (0.29)	<0.001

Abbreviations: ABI (ankle–brachial index), BMI (body mass index), SD (standard deviation).

**Table 2:** Ankle–brachial index defined hemodynamic severity in patients with and without inter-leg ABI asymmetry.

	No Inter-Leg ABI Asymmetry (*n* = 2647)	Inter-Leg ABI Asymmetry (*n* = 666)	*p* Value
Mild PAD (ABI 0.70–0.89)	2456 (92.8%)	385 (57.8%)	<0.001
Moderate PAD (ABI 0.40–0.69)	172 (6.5%)	240 (36.0%)	<0.001
Severe PAD (ABI < 0.40)	19 (0.7%)	41 (6.2%)	<0.001

**Table 3:** Events over 2 years of follow-up.

	No Inter-Leg ABI Asymmetry (*n* = 2647)	Inter-Leg ABI Asymmetry (*n* = 666)	*p*-Value
Major amputation, *n* (%)	48 (1.8%)	68 (10.2%)	<0.001
Need for revascularization, *n* (%)	108 (4.1%)	143 (21.5%)	<0.001
MALE composite (major amputation or need for revascularization), *n* (%)	131 (4.9%)	173 (26.0%)	<0.001

Abbreviations: ABI (ankle–brachial index), MALE (major adverse limb event).

**Table 4:** Multivariable analysis of the association between inter-leg ankle–brachial index asymmetry and adverse limb events over 2 years of follow-up.

	Adjusted OR *	95% CI Low	95% CI High	*p*-Value
Major amputation	4.44	2.88	6.84	<0.001
Need for revascularization	3.06	2.25	4.16	<0.001
Composite MALE outcome (major amputation or need for revascularization)	3.70	2.80	4.91	<0.001

* Adjusted for age, sex, body mass index, and peripheral artery disease severity based on the ankle–brachial index. Abbreviations: MALE (major adverse limb event), OR (odds ratio), CI (confidence interval).

## Data Availability

The original contributions presented in the study are included in the article; further inquiries can be directed to the corresponding author.
